# Virulence of *Vibrio alginolyticus* Accentuates Apoptosis and Immune Rigor in the Oyster *Crassostrea hongkongensis*

**DOI:** 10.3389/fimmu.2021.746017

**Published:** 2021-09-21

**Authors:** Fan Mao, Kunna Liu, Nai-Kei Wong, Xiangyu Zhang, Wenjie Yi, Zhiming Xiang, Shu Xiao, Ziniu Yu, Yang Zhang

**Affiliations:** ^1^Chinese Academy of Sciences Key Laboratory of Tropical Marine Bio-resources and Ecology and Guangdong Provincial Key Laboratory of Applied Marine Biology, South China Sea Institute of Oceanology, Chinese Academy of Science, Guangzhou, China; ^2^Southern Marine Science and Engineering Guangdong Laboratory (Guangzhou), Guangzhou, China; ^3^College of Earth and Planetary Sciences, University of Chinese Academy of Sciences, Beijing, China; ^4^Department of Pharmacology, Shantou University Medical College, Shantou, China

**Keywords:** *Vibrio alginolyticus*, oyster, virulence, host-pathogen interactions, proteomics

## Abstract

*Vibrio* species are ubiquitously distributed in marine environments, with important implications for emerging infectious diseases. However, relatively little is known about defensive strategies deployed by hosts against *Vibrio* pathogens of distinct virulence traits. Being an ecologically relevant host, the oyster *Crassostrea hongkongensis* can serve as an excellent model for elucidating mechanisms underlying host-*Vibrio* interactions. We generated a *Vibrio alginolyticus* mutant strain (*V. alginolyticus* ^△^ *^vscC^*) with attenuated virulence by knocking out the *vscC* encoding gene, a core component of type III secretion system (T3SS), which led to starkly reduced apoptotic rates in hemocyte hosts compared to the *V. alginolyticus* ^WT^ control. In comparative proteomics, it was revealed that distinct immune responses arose upon encounter with *V. alginolyticus* strains of different virulence. Quite strikingly, the peroxisomal and apoptotic pathways are activated by *V. alginolyticus* ^WT^ infection, whereas phagocytosis and cell adhesion were enhanced in *V. alginolyticus* ^△^ *^vscC^* infection. Results for functional studies further show that *V. alginolyticus* ^WT^ strain stimulated respiratory bursts to produce excess superoxide (O2^•−^) and hydrogen peroxide (H_2_O_2_) in oysters, which induced apoptosis regulated by p53 target protein (p53tp). Simultaneously, a drop in sGC content balanced off cGMP accumulation in hemocytes and repressed the occurrence of apoptosis to a certain extent during *V. alginolyticus* ^△^ *^vscC^* infection. We have thus provided the first direct evidence for a mechanistic link between virulence of *Vibrio* spp. and its immunomodulation effects on apoptosis in the oyster. Collectively, we conclude that adaptive responses in host defenses are partially determined by pathogen virulence, in order to safeguard efficiency and timeliness in bacterial clearance.

## Introduction

*Vibrio alginolyticus* is a Gram-negative bacterium ubiquitously found in aquatic and marine habitats and poses considerable health risks to marine animals and humans alike *via* the local food-chains (1). Human infections by *V. alginolyticus* occur primarily through consumption of insufficiently cooked aquatic food products, with consequences such as diarrhea, gastroenteritis, and septicemia (2). More importantly, *V. alginolyticus* has been recognized as a dominant causative agent of lethal outbreaks of vibriosis in many marine species (3–6). For example, the embryonic oyster is vulnerable to vibriosis, which causes massive mortality of larvae and thus heavy losses to the aquaculture industry (7–9).

Increasingly, economic casualties and health risks annually brought about by these pathogenic bacteria have aroused global concerns. In recent years, research on the pathogenic mechanisms of *V. alginolyticus* has gained momentum, which enables the identification of some key virulence factors including polar and lateral flagella, hemolysins, alternative sigma factor RpoX, outer membrane protein A (OmpA), type III secretion system, and so on (3, 10, 11). Among this, type III secretion system (T3SS) of *V. alginolyticus* has emerged as an important virulence determinant, whereby variable effectors are directly translocated into the host cytosol for manipulating cellular responses (10, 12–14). For its relatively high pathogenic potential, the need to develop vaccines, which immunologically target this pathogen in prophylactic applications, is particularly urgent. In this light, the construction of knockout mutant strains such as HY9901△*tyeA* and △*clpP* provided evidence for attenuated live vaccines in fish and shrimp (15, 16). Thus far, however, a gap remains in our mechanistic understanding of how the host lacking adaptive immunity responds to *Vibrio* with distinct virulence profiles.

The Hong Kong oyster (*Crassostrea hongkongensis*) is an edible bivalve. As a commercially valuable species, it is endemic to the estuarine and coastal regions of the South China Sea, with an aquaculture history of more than 700 years (17). Within its native habitat, *C. hongkongensis* is constantly exposed to microbial assaults in a dynamic intertidal environment (18, 19). Its ability to survive in such extreme environments is dependent on an open yet sophisticated circulation system that executes innate immune functions and allows unique strategies for stress adaptation, making oysters suitable biomarkers for health assessment of marine ecosystems (20, 21). As adept phagocytes, oyster hemocytes function in both cellular and humoral defenses, which provide a fundamental basis for oysters to cope with infectious agents (22, 23). Through transduction of immune signals, these circulating hemocytes recognize self and non-self-parts, while undergoing agglutination, phagocytosis, and encapsulation processes. Elimination of microbial pathogens is accomplished by coordinated actions of released reactive oxygen species (ROS), various hydrolytic enzymes, lysosomes, and cell apoptosis to maintain homeostasis systems (24). It was recently revealed that *Vibrio* species such as *Vibrio tasmaniensis* and *Vibrio crassostreae* of different cytotoxic potential adopt species-specific strategies to infect oysters, resulting in variable infection outcomes (25). This inspired us to explore the oyster host interactions in response to differentially virulent pathogens and the molecular underpinnings of host defenses therein.

Previously, a *V. alginolyticus* strain Zjo5 (*V. alginolyticus* ^WT^) was isolated from a marine source, which has a capacity for inducing apoptosis in host hemocytes (14). We characterized the oyster hemocytes’ fates after infection by *V. alginolyticus* ^Zjo5^ with distinct virulence traits, by using the wide-type strain *V. alginolyticus* ^WT^ and the attenuated strain *V. alginolyticus* ^△^ *^vscC^*. The attenuated strain *V. alginolyticus* ^△^ *^vscC^* was constructed by mutation of *vscC* fragment in the *V. alginolyticus* ^WT^ genome, which constitutes part of the core components of T3SS (26). Through a proteomic approach, we determined differentially expressed proteins (DEPs) in response to infection by different virulent *Vibrio* strains, and analyzed the trends of how such DEPs regulate immune outcomes in oysters. While it is known that distinct mechanisms arise independently during mollusk evolution, few studies have advanced a detailed mechanistic view on interactions between mollusk host and *Vibrio alginolyticus* with different virulence profiles. Here, we provided the first direct and specific evidence on the molecular basis for underlying virulence-related immunomodulation of mollusk host response during infection by *V. alginolyticus*.

## Materials and Methods

### Animals Acclimation

Two-year-old *C. hongkongensis* (shell length 100 ± 10 mm) were collected from Zhanjiang, Guangdong Province, China, and acclimated to laboratory conditions by rearing in aerated sand-filtered seawater at ambient temperature (27 ± 1°C) and appropriate salinity (25‰) for 2 weeks prior to experiments. During the acclimation period, oysters were fed with the microalgae *Isochrysis galbana* (10^5^ cells/ml) and *Chaetoceros calcitrans* (2 × 10^5^ cells/ml) twice a day.

### Flow Cytometric Assay on Apoptotic Cells Following *Vibrio* Challenge

For flow cytometric analysis on apoptosis, oysters in the challenged groups were injected with 100 μl *V. alginolyticus* ^WT^ or *V. alginolyticus* ^△^ *^vscC^* (suspended in 0.1 M PBS at a density of 1.0 × 10^9^ cells/ml) into the adductor muscle, respectively. The control group was injected with sterile PBS of the same volume (pH 7.4). After 30 min, oysters were returned to water tanks, and their hemocytes were randomly collected at 12 h post-injection. Three biological replications were performed in each assay, except for five replications for RNA interference assay. Then, hemocytes were incubated with 5 μl Annexin V-FITC and 5 μl propidium iodide (PI) supplied in Annexin V-FITC/PI apoptosis detection kit (Vazyme, A211, Nanjing, China) at room temperature (10 min in dark). Next, 400 ml binding buffer was added to the suspension, before analysis by a Guava^®^ easyCyte™ flow cytometer (Millipore, USA). At least 10,000 cell events were gated. Statistical analysis was performed with FlowJo (v10.0 software). Annexin V-FITC could bind to phosphatidylserine (PS) and indicate early apoptosis event, whereas PI only binds to exposed DNA in late apoptotic cells or necrosis cells. Hence, the early apoptotic cells were stained only by PS and detected in lower right area (FITC^+^/PI^−^) in flow cytometry dot chart (Q3). The late apoptotic cells were in the upper right area co-stained with FITC and PI (FITC^+^/PI^+^, Q2). The non-living or necrosis cells were in the upper left area (FITC^−^/PI^+^, Q1). Living cells cannot be stained, neither Annexin V-FITC nor PI, which will be displayed in the lower left corner (FITC^−^/PI^−^, Q4).

### Bacterial Clearance Assay

For this assay, similar challenge strategies were applied, and hemocytes were randomly sampled at 0, 6, 12, and 24 h post-injection for bacterial clearance assay. Three oysters were pooled as one sample, and three samples were collected at each time point. Hemocytes harvested from the oystersin, the *V. alginolyticus* ^WT^ and *V. alginolyticus* ^△^ *^vscC^* treated groups, were centrifuged at 400 × g at 4°C for 3 min and washed by PBS three times. Subsequently, hemolymph was lysed in 200 µl cold PBS containing 0.05% Triton X-100 for 1 h. Lysates from each tube (10 µl) were serially diluted in PBS, from which 100 µl lysates was spread on LB plates followed by overnight culture for enumeration of bacterial colony counts to determine bacterial clearance efficiency.

### Protein Extraction, Digestion, and IBT Labeling

Hemolymph were collected from the PBS-, *V. alginolyticus* ^WT^-, and *V. alginolyticus* ^△^ *^vscC^*-injected groups for protein extraction. Three samples per group were collected as biological replicates. Hemocytes were centrifuged and washed three times with PBS followed by incubation in a lysis buffer containing 1 mM phenylmethylsulfonyl fluoride and 2 mM ethylenediaminetetraacetic acid for 5 min on ice. Then, the lysate was sonicated for 5 min (2 s/3 s) after adding 10 mM dithiothreitol (DTT), and centrifuged at 25,000 × g for 20 min at 4°C. Five times the volume of pre-cooled acetone was added to supernatant and incubated for 2 h at 20°C to extract total proteins. The precipitates were redissolved in a lysis buffer and prepared for quantification by using the Bradford method. Subsequently, 100 μg of total protein per sample were digested by adding 2.5 μg trypsin enzyme at 37°C for 12 h. Then, enzymatic peptides were desalted by Strata X column and vacuum drying.

An IBT reagent 10-plex kit (Pulijian Company, Nanjing, China) was used to perform isobaric tagging according to the manufacturer’s protocol. Briefly, 2 mg of IBT reagent was dissolved in 80 μl isopropanol. Peptides (100 μg) were reconstituted in 0.2 M TEAB to a final concentration of 4 μg/μl and rapidly mixed with the 80 μl IBT reagent followed by incubation at room temperature for 2 h to be well labeled.

### Peptide Fractionation and LC-MS/MS Analysis

Shimadzu LC-20AB liquid phase system was applied for separating peptides coupled with a 5 μm 4.6×250 mm Gemini C18 separation column (Shimadzu, Kyoto, Japan). Extracted peptides were reconstituted with 2 ml mobile phase A (5% ACN, pH 9.8), and eluting at a flow rate of 1 ml/min with gradient: 5% mobile phase B (95% ACN, pH 9.8) for 10 min, 5 to 35% mobile phase B for 40 min, 35 to 95% mobile phase B for 1 min, mobile phase B lasting for 3 min, and 5% mobile phase B for 10 min for equilibrium. Peptide samples were collected at 214 nm at every min followed by freeze-drying. Afterwards, the extracted peptides were reconstituted again with 2 ml mobile phase A (5% ACN, pH 9.8), for high-performance liquid chromatography (HPLC) with Shimadzu LC-20AD nanoliter liquid chromatograph. Peptides separated by a nanoliter liquid-phase system were then applied for identifying by mass spectrometer.

Subsequently, separated peptides were directly interfaced with a Thermo Q Exactive Benchtop mass spectrometer (Thermo Fisher Scientific, San Jose, CA, USA) for first- and second-order mass spectrometric analyses. The main parameters are set as follows: electrospray voltage, 1.6kV; the first-order MS/MS scan, from 350 to 1,600 m/z at resolution of 70,000; the second-order MS/MS scan, 100 m/z at resolution of 17,500. Ion fragmentation was filtrated with charge from +2 to +7 and peak intensity >10,000 by using a high-energy collision-induced dissociation (HCD) dual-scan approach.

### Protein Identification and Quantification

Raw LC-MS/MS data were converted to MGF files by using Proteome Discoverer (Thermo scientific). Proteins were identified by using Mascot search engine version 2.3.02 (Matrix Science, London, UK). The software IQuant (BGI, Shenzhen, China) was used to quantitatively analyze peptides labeled with IBTs. For false discovery rate (FDR) calculations, an automatic decoy database search strategy was employed to estimate FDR by using a Prcolator algorithm. Proteins containing at least one unique set of spectra with filtration of FDR<=1% were used for follow-up quantification analysis. A volcano-gram was drawn to filter differentially expressed proteins (DEPs) between the PBS-, *V. alginolyticus* ^WT^-, and *V. alginolyticus* ^△^ *^vscC^*-injected groups.

### Bioinformatics Analysis

Protein annotations were predicted by using the Blast2GO tool in the Gene Ontology (GO) platform (http://www.geneontology.org/). The GO enrichment was performed by using annotation of differentially expressed proteins (DEPs) with a hypergeometric test. Pathway enrichment analysis is useful for determining main metabolic pathways and signaling pathways implicating identified proteins. This was done by using a search pathway tool in the KEGG Mapper platform (http://www.genome.jp/kegg/mapper.html). Heatmaps were organized to display the expression level of DEPs in each group by TBtools software (27).

### Measurement of O2^•−^ and H_2_O_2_

For superoxide $\left({\text{O}}_{2}^{•-}\right)$ detection, working solutions of 5 μM MitoSOX™ reagent (Invitrogen, M36008, USA) were prepared in Ca^2+^- and Mg^2+^-supplemented Hank’s balanced salt solution (HBSS; BBI, E607006) and used to incubate hemocytes for 10 min at 37°C, shielded from light. Hemocytes were harvested after bacterial exposure to oysters *in vivo*. Three oysters were pooled as one sample, and three biological samples were collected. Finally, hemocytes were washed and resuspended in fresh HBSS for detection of mitochondrial ${\text{O}}_{2}^{•-}$ by flow cytometry. At least 10,000 cell events were gated. Statistical analysis was performed with FlowJo (v10.0 software).

Hydrogen peroxide was measured by the Amplex Red hydrogen peroxide/peroxidase assay kit (Invitrogen, A22188, USA) according to the manufacturer’s instructions. Briefly, oyster hemocytes were collected and mixed isometrically in a working solution containing 10 U/ml horseradish peroxidase and 10 mM Amplex Red. The mix was incubated at room temperature for 30 min, shielded from light. Absorbance at 560 nm was determined by using Sunrise microplate reader (Tecan, Switzerland).

### Hydrogen Peroxide Stimulation

Hydrogen peroxide (H_2_O_2_ 3% w/v; Sigma, 7722-84-1, USA) was applied to induce oyster hemocyte apoptosis *in vitro*. MTT [3-(4,5-dimethylthiazol-2-yl)-2,5-diphenyltetrazolium bromide] assay was performed to analyze cell viability and determine the appropriate H_2_O_2_ concentrations for hemocytes treatment, according to previous reports (28). Then, 0.1 mM and 1.0 mM H_2_O_2_ was applied to incubate the hemocytes for 1.5 h followed by their collection for RNA extraction and apoptosis analysis. Three oysters were pooled as one sample, and three biological samples were collected.

### Knockdown of Chp53 Target Protein *In Vivo*

The cDNA fragments were amplified with primers paired with T7 promoter overhangs by using a Promega RiboMAX™ Express RNAi system. Similarly, a GFP cDNA fragment was amplified to serve as an experimental control. PCR products were used as templates to synthesize dsRNA. Forty oysters were randomly assigned into four groups and placed into four tanks: dsGFP + PBS, dsGFP + *V. alginolyticus* ^WT^, dsp53tp + PBS, dsp53tp + *V. alginolyticus* ^WT^. Each oyster was injected with 50 μg dsRNA. Three days after injection, five individuals from each group were picked randomly for collection of hemocytes to obtain biological replicates. These samples were immediately centrifuged (2,000 rpm/min for 3 min at 4°C) to harvest hemocytes followed by RNA extraction and apoptosis analysis.

### Quantitative Real-Time PCR

Hemocytes after H_2_O_2_ stimulation and *Ch*p53-target protein-depletion were applied for total RNA extraction by using TRIzol reagent (Invitrogen). Total RNA extracted were then applied for the first-strand cDNA synthesis followed by qRT-PCR with specific primers (**Supplementary Table 1**). Each assay was performed in triplicates with GADPH mRNA as an internal control. Moreover, qRT-PCR was conducted by using a Light Cycler 480 (Roche) in a reaction volume of 20 μl containing 1 μl of template cDNA, 10 μl of 2× SYBR green mix, 0.5 μl of each primer (10 pmol/μl), and 8 μl of PCR-grade water. Analysis on the dissociation curve of the amplification products was performed to verify specificity at the end of each PCR. The relative expression of *Ch*P53 target protein was calculated by using the 2^−ΔΔCT^ method (29).

### Flow Cytometric Determination of Apoptotic Cells Induced by Hydrogen Peroxide, p53-Target Protein-Depletion, and cGMP

Hemocytes were collected and resuspended in 100 μl binding buffer. Then, staining (10 min in dark) was performed at room temperature with 5 μl Annexin V-FITC and 5 μl propidium iodide (PI) supplied by an apoptosis detection kit (Vazyme, A211). Next, another 400 μl binding buffer was added to the suspension, before analysis by a Guava^®^ easyCyteTM flow cytometer (Millipore). At least 10,000 cell events were gated. Statistical analysis was performed with FlowJo (v10.0 software). Similarly, hemocyte apoptosis was analyzed after p53 target protein depletion and cGMP injection. Three biological replications were performed in each assay, except for five replications for RNA interference assay.

### Measurement of Intracellular cGMP Post-Infection

For cGMP assay, oyster hemocytes were collected in 1.5 ml centrifuge tubes after *in vivo* infection, following by centrifugation and resuspension in PBS. Three oysters were pooled as one sample and three biological samples were collected. Then, cells were subjected to repeated freeze-thaw cycles in liquid nitrogen until all cells were lysed. cGMP was measured by a cGMP ELISA detection kit (Meimian, MMJZ-9507701, China) according to the manufacturer’s instructions. Briefly, cell lysates were loaded into microplates followed by addition of 50 μl HRP-conjugated reagent, covering the plates with a plate sealer and incubating the samples at 37°C for 60 min. Subsequently, a 1× washing buffer was used to wash the plates four times. The plates were processed to remove residual liquid in the wells after wash step. For cGMP/HRP-cGMP incubation, 50 μl assay buffer A and 50 μl assay buffer B were added to the wells and incubated at 37°C for 30 min in dark. Next, 50 μl of the stop solution was added to all wells to stop enzymatic reactions followed by data acquisition with the plates on a microtiter plate reader at 450 nm. Quantitative measurements were obtained against a standard curve.

### cGMP Stimulation

cGMP injection experiments were performed as described previously (30). To test the effects of exogenous cGMP (Sigma-Aldrich, G7504, USA) on apoptosis, each oyster was injected with 100 mM cGMP *via* the adductor muscle. The control group was injected with sterile PBS (pH 7.4; Sango Biotech, China) of the same volume. After 30 min, oysters were returned to water tanks, and hemocytes were randomly collected at 6 h post-injection for apoptotic detection assays. Three oysters were pooled as one sample, and three biological samples were collected.

## Results

### Effects of Challenges by Differentially Virulent *V. alginolyticus* Strains on Host Immune Cells

**Figure 1:**
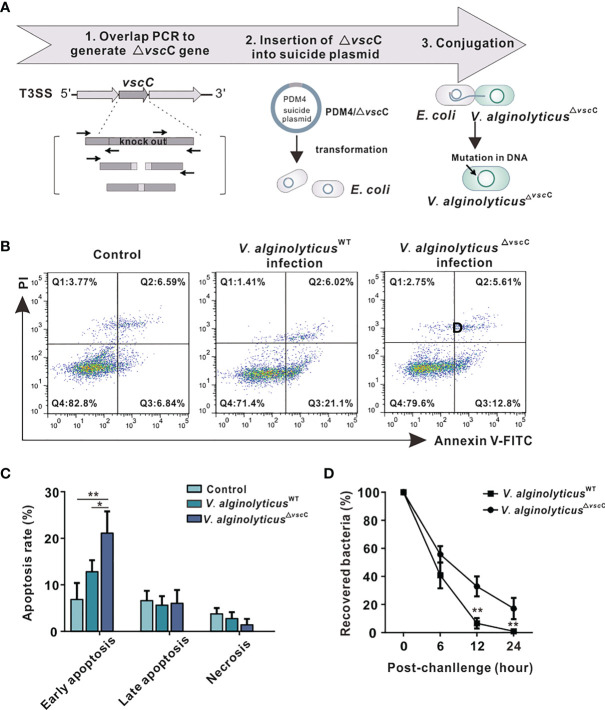
Effects of challenges by differentially virulent *V. alginolyticus* strains on host immune cells. **(A)** Workflow of the construction of attenuated *alginolyticus* ^△^ *^vscC^* strain. A *vscC*-deletion fragment was made by overlap PCR to insert it in the suicide vector pDM4, generating pDM4/△*vscC*. *E.coli* strains (SY327 and S-17) were respectively transformed with the recombinant suicide plasmid. Subsequently, conjugative transfer with *V. alginolyticus* ^WT^ was performed to obtain the mutant *V. alginolyticus* ^△^ *^vscC^* strain. **(B)** Representative flow cytometry results for apoptosis of *C. hongkongensis* hemocytes following double staining with annexin V-fluorescein isothiocyanate and propidium iodide. Q1: Cells stained as PI positive only (upper left quadrant) were necrotic/non-viable cells. Q2: Cells stained doubly positive by annexin V-FITC and PI (top right quadrant) were cells undergoing late apoptosis. Q3: On the lower right quadrant were cells stained as annexin V-FITC positive only, corresponding to cells undergoing early apoptosis. Q4: The lower left quadrant (PI and annexin V negative cells) shows living cell population. **(C)** Statistical analysis of wild-type strain and attenuated strain-induced apoptosis, including early apoptosis, late apoptosis, and necrosis. Data were analyzed in GraphPad 8.0 by two-way ANOVA and presented as mean ± SEM (n = 3). Statistical significance was determined at *p < 0.05 and **p < 0.01. **(D)** Bacterial clearance of oyster hemocytes following *V. alginolyticus* ^WT^ and *V. alginolyticus* ^△^ *^vscC^* injections (injected with a concentration of 10^9^ CFU/ml). The *x*-axis displays time lapsed post-injection, and the *y*-axis shows bacterial clearance efficiency (recovered bacteria). Data were analyzed in GraphPad 8.0 by two-way ANOVA (n = 3). Statistical significance was determined at *p < 0.05 and **p < 0.01.

The *V. alginolyticus* ^△^ *^vscC^* mutant strain was constructed as previously described (14), whose workflow is as summarized in **Figure 1A**. Briefly, an in-frame deletion was made by overlap PCR to construct a mutant of the *vscC* gene in the *Zjo5* strain. The resultant fragment lacked all coding sequences of the *vsc*C gene and was inserted in the suicide vector pDM4 to generate pDM4/△*vscC*. *E. coli* (SY327 and S-17) was transformed with this recombinant suicide plasmid. Subsequently, conjugative transfer with *V. alginolyticus* ^WT^ was performed. Attenuated strain *V. alginolyticus* ^△^ *^vscC^* was selected on TCBS+Cm plates followed by a 10% sucrose selection process. Immunofluorescent staining was carried out to confirm the loss of protein secretion function in the mutant strain (*V. alginolyticus* ^△^ *^vscC^*).

To characterize how virulence of the wild-type and attenuated strains influences host immune response, apoptosis assay was performed in oysters. The results show that *V. alginolyticus* ^WT^ infection induced a higher level of hemocyte apoptosis, compared with infection by the attenuated strain *V. alginolyticus* ^△^ *^vscC^* or treatment with PBS control (**Figure 1B**). Statistical analysis (**Figure 1C**) was conducted to provide further details on early apoptosis, late apoptosis, and necrosis in each group. Significant differences could only be observed in early apoptosis, whose rate in the *V. alginolyticus* ^WT^ infection group (21.10%) was significantly higher than that in the *V. alginolyticus* ^△^ *^vscC^* group (12.80%, *p <*0.05) and control group (6.84%, *p* < 0.01). Percentage of late apoptosis and necrosis cells had no significant differences among the three groups. Next, to investigate to what extent bacterial virulence impacts host immune outcomes, bacterial clearance assay was performed. Oyster hemocytes were lysed at 0, 6, 12, and 24 h post infection, and cultured on LB plates. As shown in **Figure 1D**, *in vivo* bacterial population was decimated over time post-infection, while the recovered bacterial in *V. alginolyticus* ^△^ *^vscC^* infection group and *V. alginolyticus* ^WT^ infection group displayed significant difference at 12 and 24 h post injection. Our results show that the host’s ability to eliminate attenuated strains (*V. alginolyticus* ^△^ *^vscC^*) reached 95% at 12 h post-infection and 100% at 24 h post-infection, while the elimination effect on *V. alginolyticus* ^WT^ is only about 65% at 12 h post infection and 80% at 24 h post infection. Overall, *Vibrio* infection can cause significantly different levels of apoptosis in host cells, which may activate different host strategies to eliminate bacteria.

### Comparative Analysis on Protein Expression Following *V. alginolyticus^WT^* Strain and *V. alginolyticus^△vscC^* Injection

**Figure 2:**
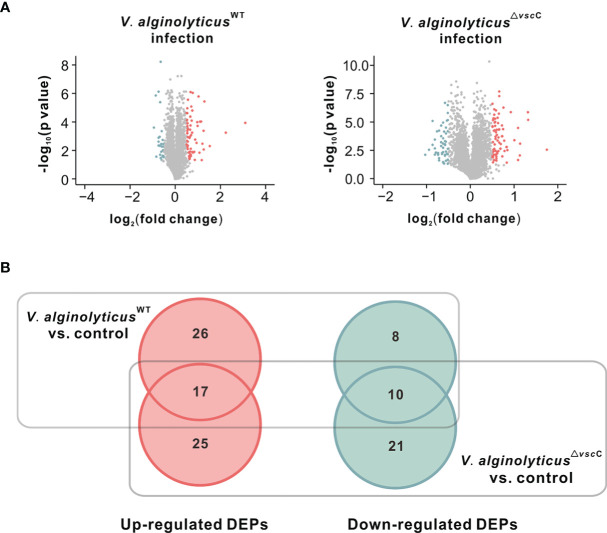
Differentially expressed proteins after injection of the *V. alginolyticus* ^WT^ strain or *V. alginolyticus^△vscC^* strain. **(A)** Volcano plots show the relationship between fold-changes and significance for the *V. alginolyticus* ^WT^ (left panel) and *V. alginolyticus* ^△^ *^vscC^* (right panel) challenged groups, compared with the uninfected control. The *y*-axis shows the -log_10_ (*p*-values), and the *x*-axis shows the difference in expression as measured in log_2_ (fold change). **(B)** Venn diagram representation of differentially expressed proteins (DEPs) following *Vibrio* injection. The numbers of specific and common DEPs for injection of the two *Vibrio* strains are as indicated. Upregulated DEPs are marked in red circles, and downregulated DEPs in green cycles.

To elucidate molecular mechanisms underlying the infection of *Vibrio* spp. of different pathogenicity, we examined protein expression patterns following challenges with *V. alginolyticus* ^WT^ or *V. alginolyticus* ^△^ *^vscC^*. IBT quantitative protein analysis produced a total of 296,436 spectra, including 43,964 unique spectra. A total of 16,510 peptides from 4,065 proteins were filtrated with FDR<=1% in total *C. hongkongensis* samples (**Supplementary Table 2**). Differentially expressed proteins were analyzed by comparing protein expression in PBS injection group with *V. alginolyticus* ^WT^ strain or *V. alginolyticus* ^△^ *^vscC^* strain challenged group, respectively. In **Figure 2A**, we use a volcano plot to summarize the magnitude, significance, and variability in oyster protein expression following challenges with *V. alginolyticus* ^WT^ or *V. alginolyticus* ^△^ *^vscC^*. Forty-three identified proteins indicate an ascending trend in protein expression, whereas 18 proteins were downregulated during *V. alginolyticus* ^WT^ infection. Meanwhile, injection with the attenuated strain *V. alginolyticus* ^△^ *^vscC^* led to the upregulation of 42 proteins and downregulation of 31 proteins. Significant protein expression in this study was defined as *p* value less than 0.05 and fold changes greater than 1.4 and below 0.7. Further details on the significantly differentially expressed proteins are provided in **Supplementary Table 3**.

A Venn diagram was plotted to analyze the specificity and common expression of proteins for *V. alginolyticus* ^WT^ stain or *V. alginolyticus* ^△^ *^vscC^* stain challenge **(** **Figure 2B**). A total of 107 proteins were differentially expressed following *Vibrio* injection, of which 68 were upregulated and 39 downregulated. Among these, 22 proteins were specifically induced, while only eight proteins were specifically downregulated following *V. alginolyticus* ^WT^ strain injection, compared with controls injected with PBS. In contrast, injection by attenuated strain *V. alginolyticus* ^△^ *^vscC^* gave rise to more specifically downregulated proteins (21 proteins) and a comparable number of specifically upregulated proteins (25 proteins). In addition, 17 upregulated and 10 downregulated proteins were found to be differentially expressed in both cases of oysters injected with *V. alginolyticus* ^WT^ strain and *V. alginolyticus* ^△^ *^vscC^*.

### Adaptive Strategies of Host Protein Expression in Response to Virulent and Attenuated *Vibrio* Strains

**Figure 3:**
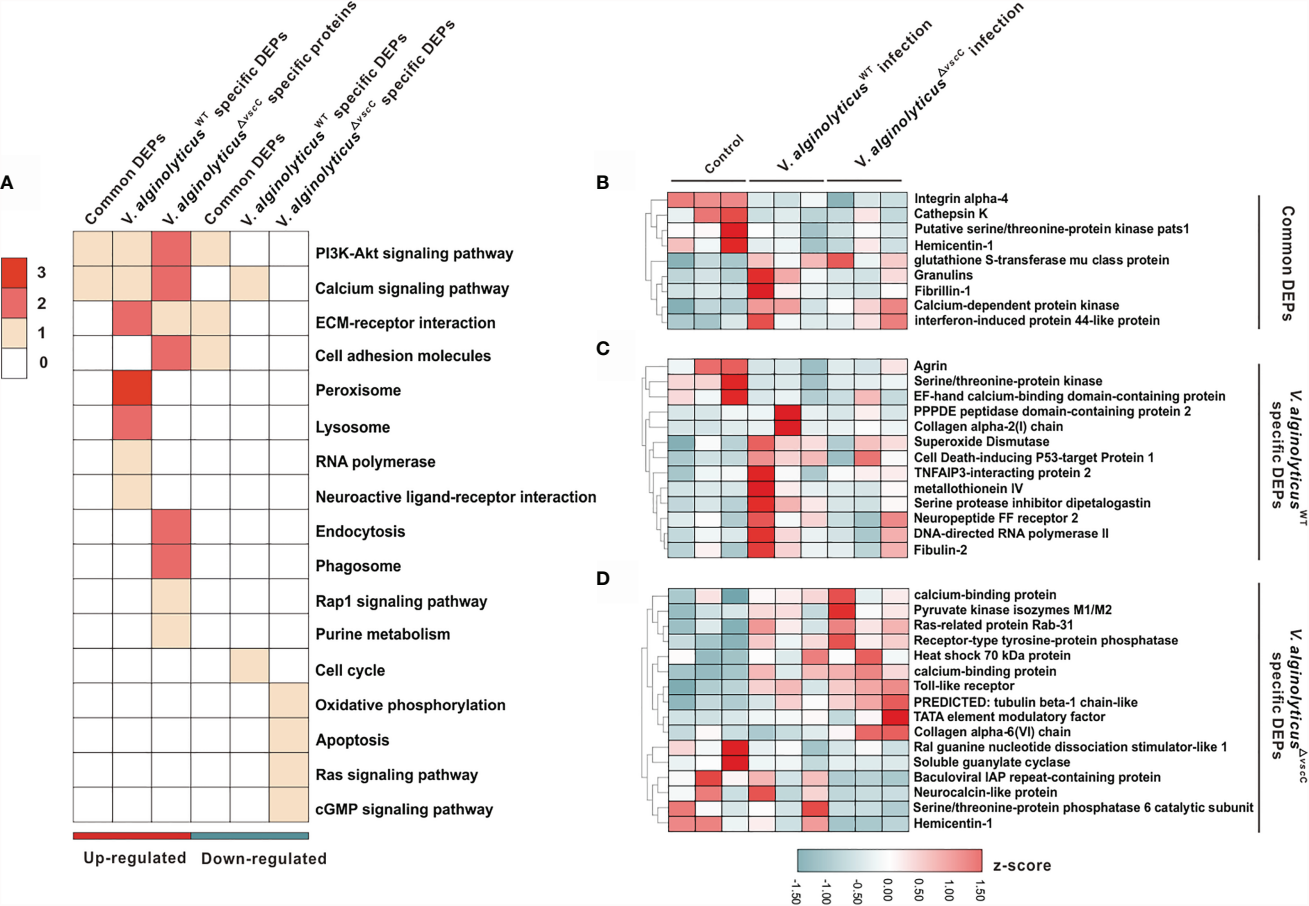
Characterization of DEPs following *V. alginolyticus*_WT_ and *V. alginolyticus* ^△^ *^vscC^* challenges. **(A)** Distribution of enriched GO terms among three clusters (common DEPs, *V. alginolyticus* ^WT^-specific DEPs, and *V. alginolyticus* ^△^ *^vscC^*-specific DEPs). The color in each term corresponds to a given protein number. **(B–D)** Heatmaps of gene expression levels of DEPs were organized according to the three clusters, Common DEPs **(B)**, *V. alginolyticus*_WT_ specific DEPs **(C)**, and *V. alginolyticus*_△vscC_ specific DEPs **(D)**. Protein expression levels were normalized by *z*-score normalization method.

To elucidate the molecular mechanisms underlying host immune defenses against *Vibrio* strains with distinct virulence profiles, GO term enrichment was performed to annotate and compare pathways induced in oysters injected with *V. alginolyticus* ^WT^ and *V. alginolyticus* ^△^ *^vscC^*. Three DEPs clusters emerged, which distinguished the host responses, including common DEPs, *V. alginolyticus* ^WT^-specific DEPs, and *V. alginolyticus* ^△^ *^vscC^*-specific DEPs. Among these, a total of 17 significant GO terms were found to be statistically enriched, which are involved in processes of host immune defenses including cell apoptosis, energy metabolism, cytoskeleton remodeling, and phagocytosis processing (**Figure 3A**). Remarkably, several signaling pathways such as peroxisomes, lysosomes, and apoptosis-related pathways were significantly and specifically induced in the *V. alginolyticus* ^WT^ infection group, whereas apoptosis and cGMP-related pathways were downregulated in the *V. alginolyticus* ^△^ *^vscC^*-challenged group. On the other hand, phagocytosis- and cell adhesion-associated pathways such as cell adhesion molecules, endocytosis, and phagosomes were enhanced in the *V. alginolyticus* ^△^ *^vscC^* challenged group.

To further understand the dynamic profiles of protein expression characteristic of the *Vibrio* strains, we analyzed 38 proteins that were differentially expressed and enriched the GO terms (**Supplementary Table 4**). Of the three clusters identified, nine proteins were collectively and differentially expressed following *V. alginolyticus* ^WT^ and *V. alginolyticus* ^△^ *^vscC^* challenges (**Figure 3B**), with respect to the control group (PBS injection). Specifically, *V. alginolyticus* ^WT^ challenge induced elevated expression of 10 proteins and blunted the expression of three proteins, namely, superoxide dismutase (SOD), metallothionein IV, and cell death-inducing p53-target protein 1, which are involved in respiratory bursts and apoptosis during infection (**Figure 3C**) (31). Protein cluster of *V. alginolyticus* ^△^ *^vscC^*-specific DEPs consists of 16 proteins, of which 10 were specifically upregulated and 6 downregulated. Notably, downregulated expression of soluble guanylate cyclase (sGC) in the cGMP signaling pathway could have an impact on intracellular cGMP levels. Interestingly, expression of Toll-like receptor 6 (TLR6) was increased significantly after *V. alginolyticus* ^△^ *^vscC^* injection, suggestive of an essential role in adaptation to *V. alginolyticus* ^WT^ challenge (**Figure 3D**).

### ROS Formation Was Accelerated During *V. alginolyticus^WT^* Infection, Promoting Hemocyte Apoptosis

**Figure 4:**
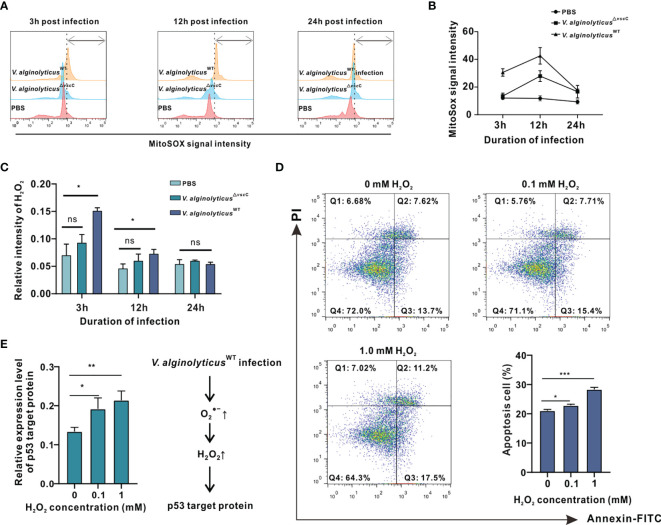
*V. alginolyticus*^WT^ challenge induced cell apoptosis and activated apoptotic pathway accompanied by intracellular ${\text{O}}_{2}^{•-}$ and H_2_O_2_ production in oyster hemocytes. **(A)** Time course of ${\text{O}}_{2}^{•-}$ release in oyster hemocytes at 3, 12, and 24 h after *V. alginolyticus* ^△^ *^vscC^* or *V. alginolyticus* ^WT^ injection, as detected in flow cytometry (FACS) analysis with the mitochondrial ${\text{O}}_{2}^{•-}$ probe MitoSOX. Red color represents the uninfected control injected with PBS; blue color represents the group injected with *V. alginolyticus* ^△^ *^vscC^*; and yellow color represents the group injected with *V. alginolyticus* ^WT^. **(B)** Quantification of MitoSOX fluorescence intensities. **(C)** Time course of H_2_O_2_ formation in oyster hemocytes at 3, 12, and 24 h after *V. alginolyticus* ^△^ *^vscC^* or *V. alginolyticus* ^WT^ injection. Data were analyzed by two-way ANOVA and presented as mean ± SEM (n = 3); *p < 0.05; ns, not significant. **(D)** Representative dot-plot results in FACS for hemocyte apoptosis following stimulation with exogenously applied H_2_O_2_ at the concentrations of 0, 0.5, and 1 mM. Q1: necrotic/non-viable cells; Q2: late apoptosis/secondary necrosis; Q3: early apoptosis; Q4: living cells. Histogram displays the statistical analysis of apoptosis in hemocytes in response to varying levels of H_2_O_2_. Data were analyzed by one-way ANOVA and presented as mean ± SEM (n = 3); *p < 0.05; ***p < 0.001. **(E)** Relative expression levels of p53 target protein (p53tp) after stimulation by exogenously applied H_2_O_2_ at the concentrations of 0, 0.5, and 1 mM. Data were analyzed by one-way ANOVA and presented as mean ± SEM (n=3); *p < 0.05; **p < 0.01. Right panel displays a presumed respiratory bursts model during hemocyte response to *V. alginolyticus* ^△WT^ infection.

In analysis on protein expression profiles, expression of proteins associated with respiratory bursts showed a clear upward trend following challenge by virulent *V. alginolyticus* ^WT^ (**Figure 3A**). Respiratory bursts are known to be a vital arm of innate immunity in phagocytes, in which antimicrobial ROS such as superoxide $\left({\text{O}}_{2}^{•-}\right)$ and H_2_O_2_ are amply produced to eliminate or degrade internalized particles and bacteria (32). To characterize host ROS production in response to challenge by the *V. alginolyticus* ^WT^ strain, we first determined relative concentrations of ${\text{O}}_{2}^{•-}$ and H_2_O_2_. Notably, mitochondrial ${\text{O}}_{2}^{•-}$ levels in the *V. alginolyticus* ^WT^ injection group rose significantly 3 h following challenge by the *V. alginolyticus* ^WT^ strain, culminated at 12 h post-challenge. In contrast, the group challenged by the attenuated *V. alginolyticus* ^△^ *^vscC^* strain did not seem to differ from the control group in terms of mitochondrial levels at 3 h **(** **Figures 4A, B** **).** However, at 12 h after challenge, the produced mitochondrial ${\text{O}}_{2}^{•-}$ content challenged by the *V. alginolyticus* ^WT^ strain seemed twice that of the attenuated *V. alginolyticus* ^△^ *^vscC^* injection group.

Next, we assessed the levels of H_2_O_2_, a common ROS downstream of ${\text{O}}_{2}^{•-}$, induced by *Vibrio* challenges (33–35). Analysis on relative fluorescence intensity corresponding to H_2_O_2_ levels reveals that challenge with the *V. alginolyticus* ^WT^ strain resulted in a significant rise in H_2_O_2_ levels at 3 and 12 h, which returned to their initial levels at 24 h (**Figure 4C**). In contrast, H_2_O_2_ levels following challenge with the *V. alginolyticus* ^△^ *^vscC^* strain did not differ from that of the control group at time points assessed. Subsequently, we examined the effects of exogenously applied H_2_O_2_ on oyster hemocytes viability. Upon treatment with H_2_O_2_ at varying concentrations, viability of hemocytes was determined by the MTT assay, which is based on absorbance measurement at 570 nm for the conversion of MTT into formazan crystals by living cells. As shown in **Supplementary Figure 1**, treatment with H_2_O_2_ above 25 mM for 2 h elicited lethal effects, whereas treatment with 0.05 to 1 mM H_2_O_2_ induced sublethal effects in hemocytes. During microbial infections, oxidative stress is one of the hallmarks of infection-induced apoptosis (36). Production of H_2_O_2_ may not only assert collateral toxic effects on host cells but also stimulate or activate the p53 signaling pathway (37). Hence, 0.1 to 1 mM H_2_O_2_ was applied as a treatment for analysis on apoptotic events in hemocytes and their gene expression patterns of p53 target protein (p53tp). Analysis on hemocyte apoptosis shows that H_2_O_2_ treatment could cause a marked increase in apoptotic cells relative to the control group. Nevertheless, the proportions of apoptotic cells (early apoptosis rate + late apoptosis rate) varied considerably in relation to the H_2_O_2_ concentration applied. The corresponding proportions of apoptosis cells were 22.86% (*p*<0.05) for 0.1 mM H_2_O_2_ treatment and 28.3% (*p*<0.001) for 1 mM H_2_O_2_ treatment (**Figure 4D**). In the meantime, we examined the effects of H_2_O_2_ treatment on the gene expression pattern of p53tp in oyster hemocytes. It has been suggested that apoptosis caused by oxidative stress may be mediated by p53-target protein such as Bak, p21^WAF1/CIP1^, mdm2, and GADD45 (30). The expression levels of p53 target protein rose significantly in response to H_2_O_2_ (in the range of 0.1 to 1 mM), supporting the involvement of p53-dependent apoptotic pathways in H_2_O_2_-induced cell apoptosis (**Figure 4E**). Overall, our data suggest that *Vibrio* infection-induced H_2_O_2_ stimulates apoptotic pathways and mobilized expression of key proteins including p53tp, during cell apoptosis.

### Chp53 Target Protein (p53tp) as a Target of Intracellular H2O2 Formation During Hemocyte Apoptosis

To better understand the mechanistic significance of H_2_O_2_-induced cell apoptosis as an event associated with *Vibrio* infection, we examined expression levels of the differentially expressed protein, *Ch*p53 target protein, post *V. alginolyticus* ^WT^ infection, which was also induced following stimulation by exogenously applied H_2_O_2_ (**Figure 4E**). Additionally, the mRNA expression level of p53tp was significantly upregulated post *V. alginolyticus* ^WT^ infection rather than *V. alginolyticus* ^△vscC^ infection, which was consistent with their protein level (**Supplementary Figure 2**), suggesting that *Ch*p53tp was only significantly upregulated post *V. alginolyticus* ^WT^ infection. Hence, we designed RNA interference assay to investigate the regulatory role of *Ch*p53tp during *V. alginolyticus* ^WT^ infection.

**Figure 5:**
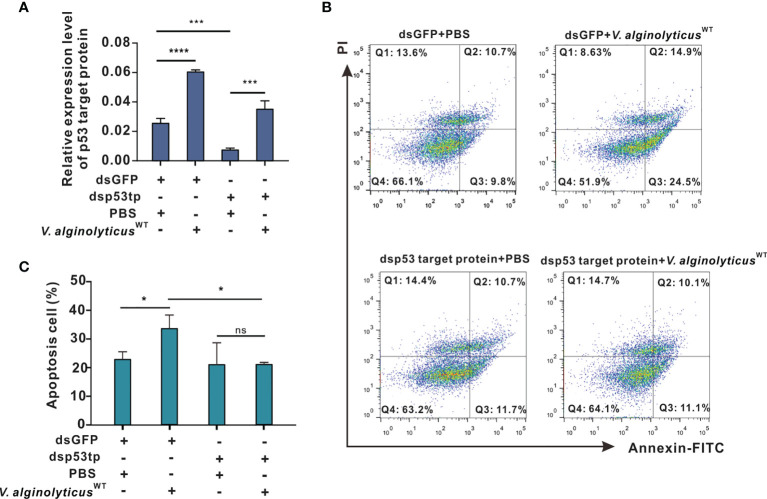
p53 target protein mediates apoptosis in oyster hemocytes. **(A)** Relative expression levels of p53 target protein after RNAi in oyster hemocytes is as shown. mRNA levels were quantified by real-time PCR with GAPDH as a reference gene. Data were analyzed by one-way ANOVA and presented as mean ± SEM (n = 5); ***p < 0.001; ****p < 0.0001. Control group: dsGFP+PBS. p53tp, p53 target protein. **(B)** Representative dot-plot results in FACS for hemocyte apoptosis after double staining with annexin V-FITC and PI. Q1: necrotic/non-viable cells; Q2: late apoptosis/necrosis; Q3: early apoptosis; Q4: living cells. **(C)** Histogram displays the statistical analysis on apoptotic cells under different treatments. Data were analyzed by one-way ANOVA and presented as mean ± SEM (n = 5), with significance being determined at *p < 0.05; ns, no significance.

Here, we cloned the open reading frame (ORF) of *Chp53 target protein*1 gene, and the deduced amino acid sequence as shown in **Supplementary Figure 3**. Domain prediction indicated a conserved LIFAT domain in the p53tp amino acid sequence as shown in **Supplementary Figure 4**. Then, an RNA interference assay was performed for *in vivo* knockdown of the expression of *Ch*p53 target protein to clarify the functional meaning of *Ch*p53 target protein as a signaling target during induction of apoptosis in oyster hemocytes. Following challenge with *V. alginolyticus* ^WT^ strain + dsGFP, expression levels of *Ch*p53 target protein were markedly upregulated by 2.4 folds (*p*<0.0001). This was significantly impaired by the injection with dsRNA of *Ch*p53 target protein, as reflected by an inhibition rate of 70% relative to the control group injected with dsGFP and PBS. Interestingly, although RNA interference by dsRNA blunted the expression of *Ch*p53 target protein, challenge with the *V. alginolyticus* ^WT^ strain still induced the expression of *Ch*p53 target protein in significant manners (dsp53tp + *V. alginolyticus* ^WT^ strain, **Figure 5A**). Accordingly, the rate of hemocyte apoptosis was significantly raised after challenge with the *V. alginolyticus* ^WT^ strain (dsGFP + *V. alginolyticus* ^WT^ strain) compared to that of the control group (dsGFP + PBS), as demonstrated earlier. However, depletion of *Ch*p53 target protein *in vivo* did not significantly affect the apoptosis rate (dsp53tp + PBS), even in the group challenged with the *V. alginolyticus* ^WT^ strain (dsp53tp + *V. alginolyticus* ^WT^; **Figure 5B**). For statistical analysis, we compared the rate of hemocyte apoptosis rate following different treatments (**Figure 5C**). The results suggest that apoptosis rate increased significantly (*p*<0.05) after challenge with the *V. alginolyticus* ^WT^ strain (dsGFP + *V. alginolyticus* ^WT^ strain), by 1.6-fold relative to the control group (dsGFP + PBS). Still, no significant difference was observed following *in vivo* depletion of *Ch*p53 target protein in hemocyte apoptosis, suggestive of the regulatory role of p53 targeting protein during *V. alginolyticus* ^WT^ infection.

### Virulence of *V. alginolyticus* Modulates Intracellular cGMP Generation and Apoptosis in Oyster Hemocytes

**Figure 6:**
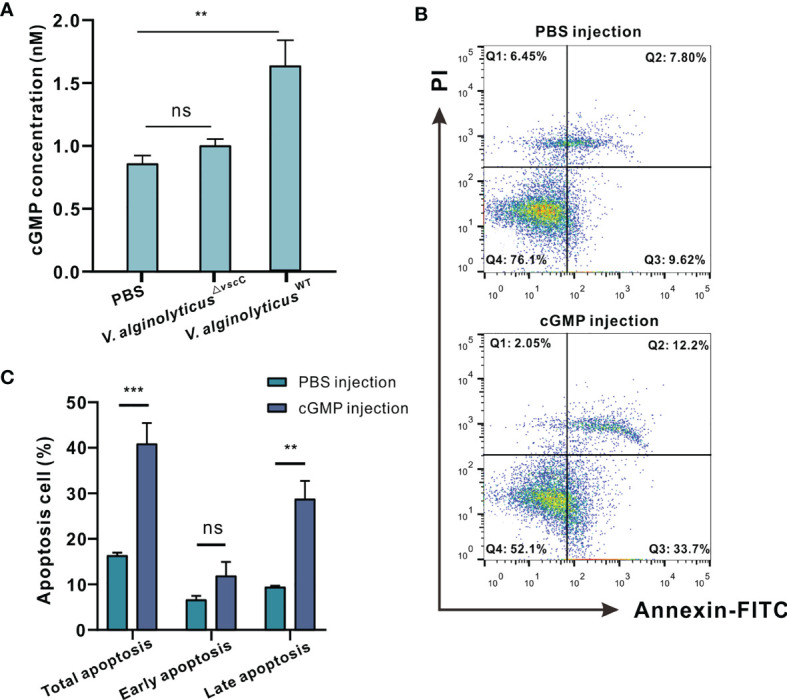
Virulence phenotypes of *V. alginolyticus* differentially modulate hemocytes apoptosis *via* cGMP generation. **(A)** Quantification of cGMP content in *C. hongkongensis* hemocytes after challenge with PBS, *V. alginolyticus* ^WT^ or *V. alginolyticus* ^△^ *^vscC^*, as determined by ELISA. cGMP concentrations were calculated by means of a standard curve as shown **Supplementary Figure 2**. Statistical difference in the extent of cGMP content of the control, *V. alginolyticus* ^WT^, and *V. alginolyticus* ^△^ *^vscC^* infection groups were determined by one-way ANOVA and presented as mean ± SEM (n = 3); ns, no significance; **p < 0.01. **(B)** Representative dot-plot results in flow cytometry (FACS) analysis on hemocytes apoptosis after PBS or cGMP injection. Q1: necrotic/non-viable cells; Q2: late apoptosis/necrosis; Q3: early apoptosis; Q4: living cells. **(C)** Statistical evaluation of the proportions of cells undergoing total apoptosis, early apoptosis, and late apoptosis after injection with PBS or cGMP. Data were analyzed by two-way ANOVA and presented as mean ± SEM (n=3); ns, no significance; **p < 0.01; ***p < 0.001.

cGMP signaling pathways were specifically enriched and downregulated following challenge with attenuated *V. alginolyticus* ^△^ *^vscC^*, as evidenced in GO term enrichment (**Figure 3A**), including soluble guanylate cyclase (sGC) as shown in **Figure 3D**. Expression level of sGC mRNA was also detected in hemocytes after *Vibrio* infection, and results showed that only *V. alginolyticus* ^△^ *^vscC^* strain but not *V. alginolyticus* ^WT^ strain infection could lead to significant downregulation of sGC (**Supplementary Figure 5**). sGC-cGMP is a pro-apoptotic pathway induced, and cGMP can promote apoptosis (38). To examine more closely the mechanisms underlying *V. alginolyticus* ^WT^-induced hemocytes apoptosis, we assessed the levels of intracellular cGMP, after infection with the *V. alginolyticus* ^WT^ strain or *V. alginolyticus* ^△^ *^vscC^* strain. ELISA analysis shows that challenge with *V. alginolyticus* ^WT^ strain considerably upregulated the levels of intracellular cGMP by 1.89-fold compared with the control group (injected with PBS), whereas no comparable changes were observed for the group challenged with *V. alginolyticus* ^△^ *^vscC^* (**Figure 6A**). Intracellular cGMP concentrations were calculated *via* a standard curve as shown in **Supplementary Figure 6**. Subsequently, exogenously applied cGMP was injected into the oysters *via* the adductor muscle to test whether excess cGMP levels would cause oyster hemocyte apoptosis. At 100 mM cGMP, apoptosis rate increased from 16.58% (early apoptosis, 9.70%; late apoptosis, 6.87%) to 41.16% (early apoptosis, 29.03%; late apoptosis, 12.13%) (**Figure 6B**). Apoptosis rate, in particular with respect to late apoptosis, was found to be significantly different following cGMP injection (*p*<0.01, **Figure 6C**). These results suggest that the virulent *Vibrio* strain induced intracellular cGMP generation, which modulated the outcomes of hemocytes apoptosis. In comparison, challenge with the attenuated strain resulted in an unaltered level of intracellular cGMP (**Figure 6A**), corresponding to the decreased expression levels of sGC in the proteome data (**Figure 3D**).

## Discussion

In this study, we have provided new evidence on how virulent traits in the strain *V. alginolyticus* ^WT^ and attenuated strain *V. alginolyticus* ^△^ *^vscC^* led to distinct outcomes in host immune response and cell death in *Crassostrea hongkongensis* hemocytes, with the virulent strain being a more potent inducer of hemocyte apoptosis. Although recent reports have proposed species-specific mechanisms of *Vibrio* cytotoxicity that enables subsequent subversion of host immunity in the oyster (39), little is known about the roles of virulence in the host-pathogen interaction processes, which motivated us to explore the potential determinants of infection outcomes. To gain mechanistic insights into immune defenses in the oyster after challenge with *Vibrio* of distinct virulence traits, we characterized the expression profiles of proteins and analyzed their changes upon exposure to the *V. alginolyticus* ^WT^ and attenuated *V. alginolyticus* ^△^ *^vscC^* strains. Subsequently, we found some activated pathways shared between the virulent and attenuated strains during *Vibrio* challenge, including PI3K-Akt signaling pathway, calcium signaling pathway, ECM-receptor interaction, and cell adhesion molecules. Previous studies have reported variance in gene expression profiles in surf clams and fish treated with two *Vibrio* species of differential pathogenicity to the host (40, 41), suggesting that distinct host strategies may be deployed against challenge by pathogens of different virulence across the animal phylum. In our proteome data, peroxisome, lysosome, and apoptosis pathway were enriched specifically during virulent strain infection. Interestingly, we showed in this study that apoptosis and cGMP signaling pathways were specifically downregulated during infection by the attenuated *V. alginolyticus* ^△^ *^vscC^* strain. The protein expression profiles of host hemocytes characterized here were indeed consistent with the virulence phenotypes of the infecting bacteria.

We further noted that enrichment of peroxisomes in GO terms could in part explain the high apoptosis rate in oyster hemocytes following injection by the virulent strain *V. alginolyticus* ^WT^. Remarkably, our analysis indicates that challenge with the virulent strain triggered off production of intracellular ${\text{O}}_{2}^{•-}$, which was involved in early apoptosis (42). Typically, EcSOD (extracellular superoxide dismutase) catalyzes the dismutation of ${\text{O}}_{2}^{•-}$ into H_2_O_2_ (43, 44), whose production was specifically activated after *V. alginolyticus* ^WT^ infection. Previous studies reported that apoptosis induced by H_2_O_2_ is regulated through control of ${\text{O}}_{2}^{•-}$ concentrations (45), with particular implications for respiratory bursts-dependent apoptosis of oyster hemocytes following virulent bacterial infection (46–48). Additionally, H_2_O_2_ production may not only have collateral toxic effects on hemocytes but also activate the p53 signaling pathway (49, 50). Remarkably, p53tp levels were found to be elevated following *V. alginolyticus* ^WT^ strain injection or H_2_O_2_ treatment. The tumor suppressor p53 functions primarily as a transcription factor (51–56). Previously, a total set of 3,661 direct p53 target genes was identified, encompassing a large spectrum of cellular responses including cell cycle arrest, DNA repair, apoptosis, metabolism, autophagy, mRNA translation, and feedback mechanisms (57). Here, p53 target protein was significantly upregulated by either *V. alginolyticus* ^WT^ infection or H_2_O_2_ treatment, rather than *V. alginolyticus* ^△vscC^ infection, strongly suggesting that virulent *Vibrio* infection or ROS boost could activate p53 signaling pathways. Moreover, the specific upregulation of p53tp mRNA after *V. alginolyticus* ^WT^ infection also supported this point. Finally, p53tp identified here was verified *via* RNA interference to regulate intrinsic apoptosis. Overall, our findings demonstrate that infection by virulent *Vibrio* strains activate respiratory bursts-dependent apoptosis in oyster hemocytes, with the induction and activation of p53 signaling pathways.

Moreover, the cGMP signaling pathway interrogated here is a putative key regulator of cell proliferation, differentiation, apoptosis, inflammation, and other processes (58, 59). sGC is the primary sensor of nitric oxide (NO), binding of which boosts the enzymatic activity (60–64). Accumulated evidence have proved that NO is produced and binding to sGC in a variety of virulent microbial infections (65–68). Thus, activation of sGC classically increases conversion of GTP to cGMP, resulting in an elevation of cGMP, which in turn initiates cGMP signaling cascades to give rise to subsequent physiological changes (69–72). In oyster, the significant decrease of sGC in *V. alginolyticus* ^△vscC^ infection group could balance the NO binding-induced activation of sGC. Additionally, nitric oxide (NO) is an induced toxic molecule post a highly virulent strain infection, causing intense cell damage, while attenuated strain infection maintained the NO level (73–75). A similar mechanism may occur in oyster infected with *V. alginolyticus* that infection with attenuated strain did not induce the production of NO, resulting in no difference in cGMP levels between *V. alginolyticus* ^△vscC^ infection and control group. Moreover, our work is consistent with previous findings that implicate activation of intracellular cGMP as an important event in cell apoptosis (76–78). Accordingly, we also provide evidence that intracellular cGMP elevations were only induced after *V. alginolyticus* ^WT^ challenge, whereas cGMP content was largely unaltered following infection by the *V. alginolyticus* ^△^ *^vscC^* strain. Taken as a whole, our data support the notion that elevations in intracellular cGMP levels elicit hemocytes apoptosis, as in the contexts of virulent *Vibrio* infection.

When external microorganisms launch invasion, oyster hemocytes respond by producing and leveraging a range of toxic metabolites (such as ROS, nitric oxide, etc.) and many lysosomal enzymes (79–81) for defense, while apoptosis is induced as a hemocyte elimination process to clear spent or infected cells. Intuitively, it would be interesting to compare this to host response during challenge of an attenuated *Vibrio* strain. After all, we observed no significant change in intracellular H_2_O_2_ level, even the apoptotic levels after *V. alginolyticus* ^△vscC^ strain infection. We anticipated a relatively mild immune mechanism, wherein Toll-like receptor 6 (TLR6) orchestrated defense strategies against infection by the *V. alginolyticus* ^△^ *^vscC^* strain through activation of downstream signaling pathways. It also seems reasonable to assume that TLR6 becomes activated during infection by the *V. alginolyticus* ^△^ *^vscC^* strain. In mammalian cells, since activation of TLRs serves to promote innate responses and initiate adaptive immunity (82, 83), such features are of interest because these pathways can be exploited as potential vaccine adjuvants (84, 85). TLR6 is located on the plasma membranes, recognizing extracellular microbial pathogenic molecules with distinct PAMPs and inducing inflammation through MyD88- and TRAF6-mediated activation of NF-κB in human (86). Previous research has also provided evidence for that TNF expression level inhibited by blocking TLRs signal in oysters when *Vibrio parahaemolyticus* invaded (87). In applied contexts, activation of TLR6 following *V. alginolyticus* ^△^ *^vscC^* strain infection in oyster hemocytes carries the implications of potential use of attenuated strains in host immune priming in an immunologically mild manner.

**Figure 7:**
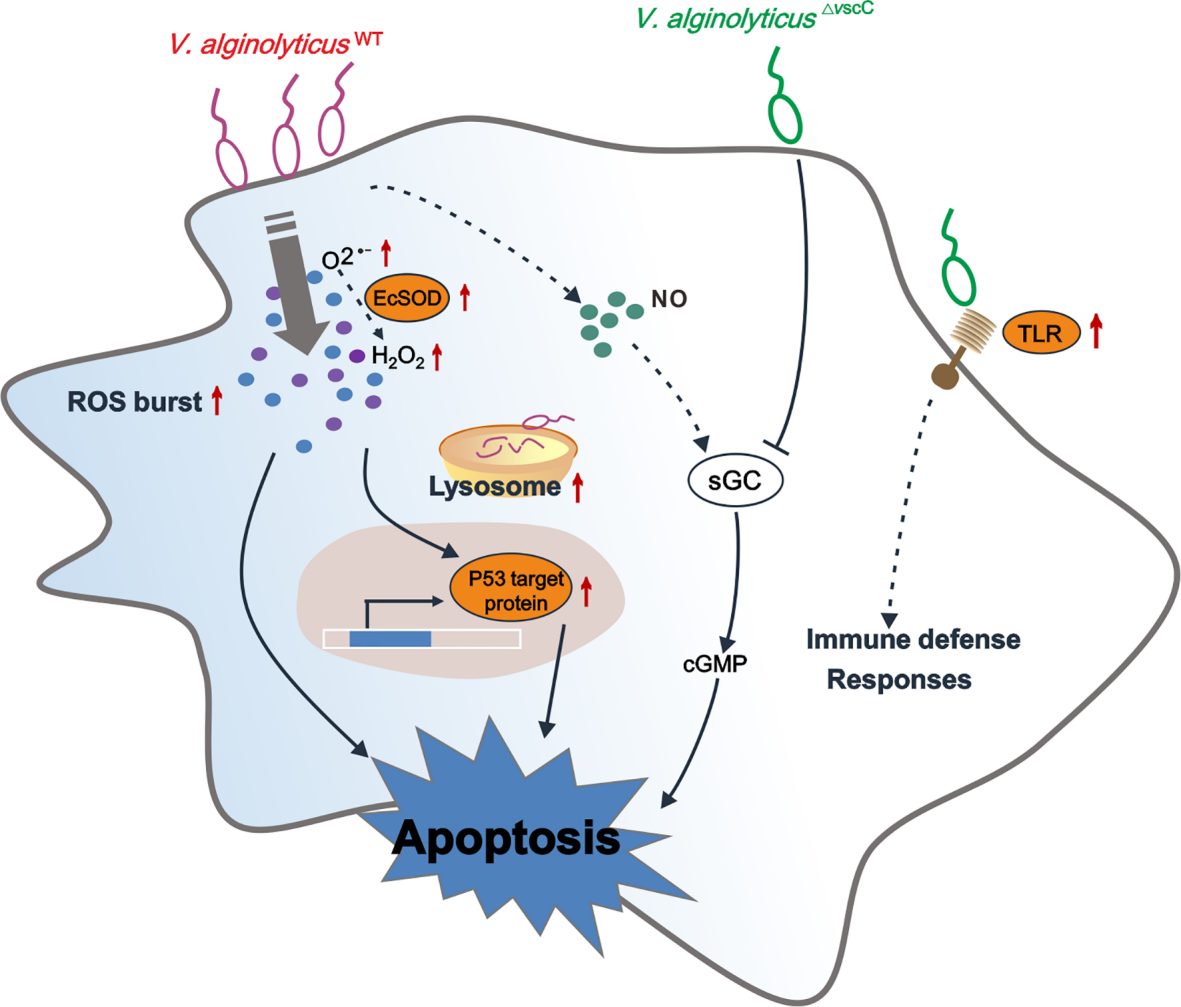
Conceptualization of mechanisms underlying immune defenses in oyster hemocytes during challenge by *Vibrio* spp. of differential virulence. *V. alginolyticus* ^WT^ challenge gives rise to accumulation of ${\text{O}}_{2}^{•-}$ and H_2_O_2_, resulting in upregulation of p53 target protein-mediated hemocyte apoptosis, along with an increase in intracellular cGMP. *V. alginolyticus* ^△^ *^vscC^* infection leads to attenuation of cellular defense of the host, along with lower levels of apoptosis. In addition, sGC-cGMP signaling pathway was specifically downregulated during attenuated *V. alginolyticus* ^△^ *^vscC^* strain infection. Interestingly, Toll-like receptor 6 were significantly upregulated during *V. alginolyticus* ^△^ *^vscC^* infection, which transduce immune signals for further elimination of pathogens through immune effector molecules, such as antimicrobial peptides (AMPs) (87, 88). Solid lines, regulatory mechanisms experimentally verified in this study; dotted lines, conjectural conclusions from the proteomic data.

In conclusion, we have interrogated the distinct outcomes of hemocytes infected by a virulent *Vibrio* strain or its attenuated counterpart in the oyster *Crassostrea hongkongensis*, which are mechanistically determined in part by the pathogen’s virulence traits. Our proteomic study described the differentially expressed proteins and enriched pathways after challenge with *Vibrio* strains of differential pathogenicity. Our findings suggest that the oyster immune system compute decisions on mild to severe responses against differentially virulent *Vibrio* strains (**Figure 7**). As a marine invertebrate model ideal for studying the interplay between environment, microbial pathogens, and host in disease dynamics (89), the oyster is useful for investigating molecular interactions between pathogenic bacteria and host cells, which in turn shed light on potential determinants of infection outcomes in metazoan hosts. We believe that attenuated pathogen strains such as that of virulent *Vibrio* species in applications deserve more attention, as immune priming in the oyster has been launched during the last few years for exploration of immunity system evolution to prevent disease outbreaks (89–93).

## Data Availability Statement

The datasets presented in this study can be found in online repositories (94, 95). The names of the repository/repositories and accession number(s) can be found below: https://www.ebi.ac.uk/pride/archive/, PXD022551.

## Author Contributions

ZY, YZ, and FM designed this study. FM, KL, and N-KW performed the data analyses and drafted the manuscript. FM, KL, and XZ conducted the experiments. WY and ZX carried out data management and constructive discussions. SX provided the experimental animals. All authors contributed to the article and approved the submitted version.

## Funding

This work was graciously supported by the National Science Foundation of China (No. 32073002, 31902404), Natural Science Foundation of Guangdong Province (2020A1515011533), Key Special Project for Introduced Talents Team of Southern Marine Science and Engineering Guangdong Laboratory (Guangzhou) (GML2019ZD0407), the Zhejiang Provincial Top Key Discipline (KF2020009), the China Agricultural Research System (CARS-49), Institution of South China Sea Ecology and Environmental Engineering, Chinese Academy of Sciences (ISEE2018PY01, ISEE2018PY03, ISEE2018ZD01), Science and Technology Planning Project of Guangdong Province, China (2017B030314052, 201707010177).

## Conflict of Interest

The authors declare that their research was conducted in the absence of any commercial or financial relationships that could be construed as a potential conflict of interest.

## Publisher’s Note

All claims expressed in this article are solely those of the authors and do not necessarily represent those of their affiliated organizations, or those of the publisher, the editors and the reviewers. Any product that may be evaluated in this article, or claim that may be made by its manufacturer, is not guaranteed or endorsed by the publisher.
