# Supplementary material for: Virulence of Vibrio alginolyticus Accentuates Apoptosis and Immune Rigor in the Oyster Crassostrea hongkongensis
Source: Front Immunol. 2021 Sep 21;12:746017. doi: 10.3389/fimmu.2021.746017 (PMC8490866; doi:10.3389/fimmu.2021.746017)
Supplement: Supplementary file 1 [file DataSheet_1.zip › supplementary material/supplementary material_R0825.docx]

***Supplementary Material***

# Supplementary Figures

##
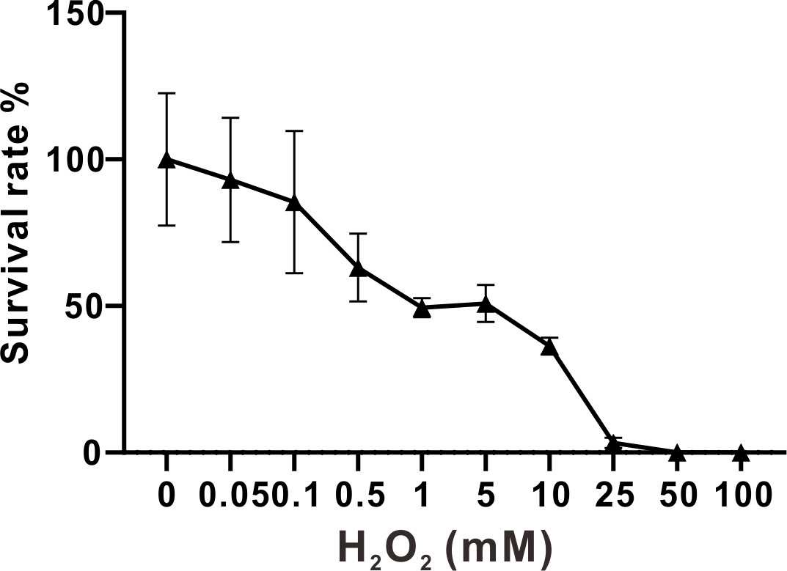


**Supplementary Figure 1.** The effects of different concentrations of H_2_O_2_ on cell viability by MTT.MTT assay was used to determine the effect of different concentration of hydrogen peroxide on the survival rate of hemocytes in oyster. The half lethal concentration reached at 1mM.


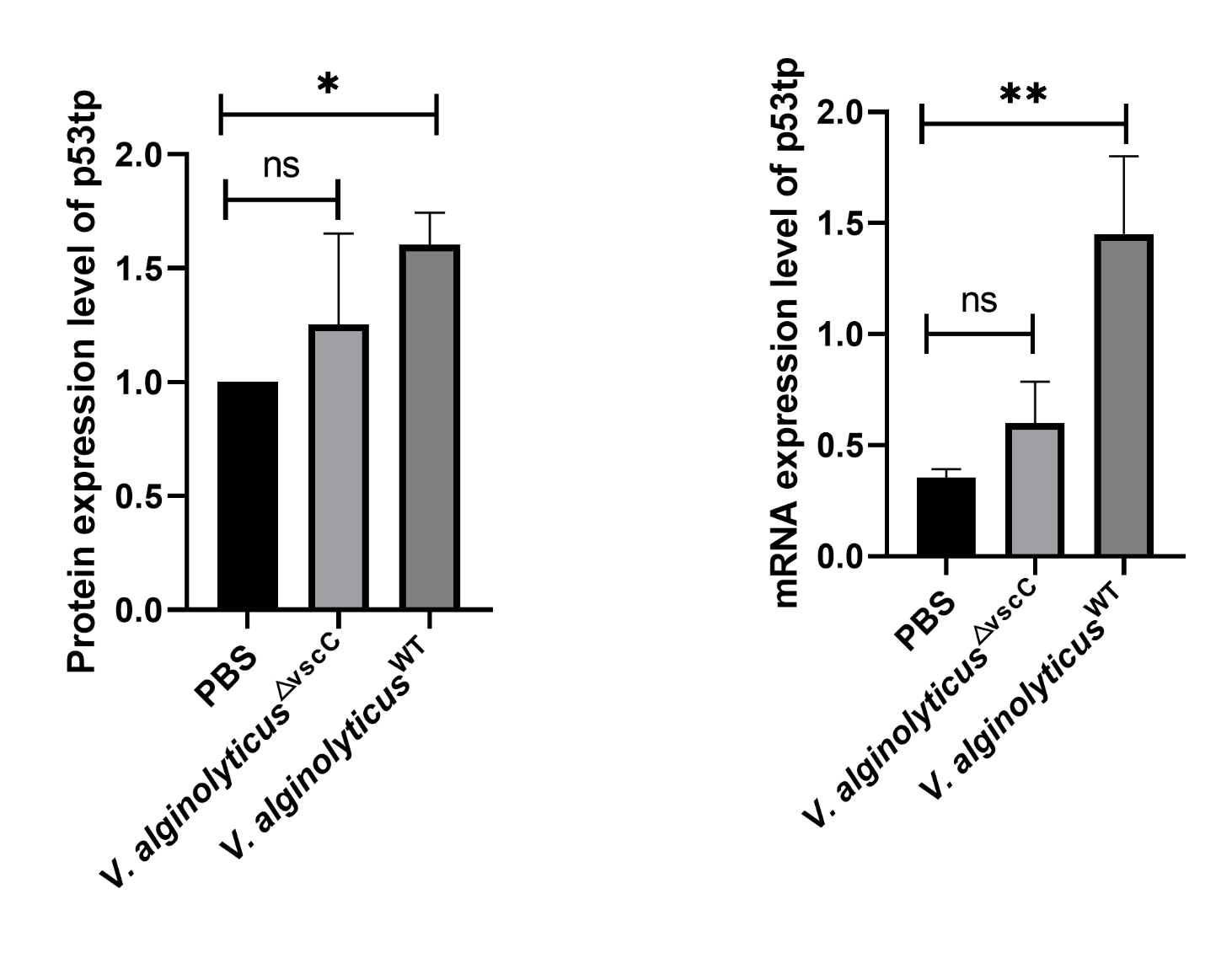


**Supplementary Figure 2. Relative expression level of *Ch*P53 target protein. (A)** Relative expression of p53 target protein at protein level. In the proteomic data, Chp53tp only significantly up-regulated post *V. alginolyticus*^WT^ infection. The expression level was set to 1 in the control group. Data were analyzed by unpaired t-test and presented as mean ± SEM (n = 3); *, p< 0.05. **(B)** Relative expression of p53 target protein at mRNA level. mRNA levels were quantiﬁed by real-time PCR with GAPDH as a reference gene. Data were analyzed by unpaired t-test and presented as mean ± SEM (n = 3); **, p< 0.01.

**
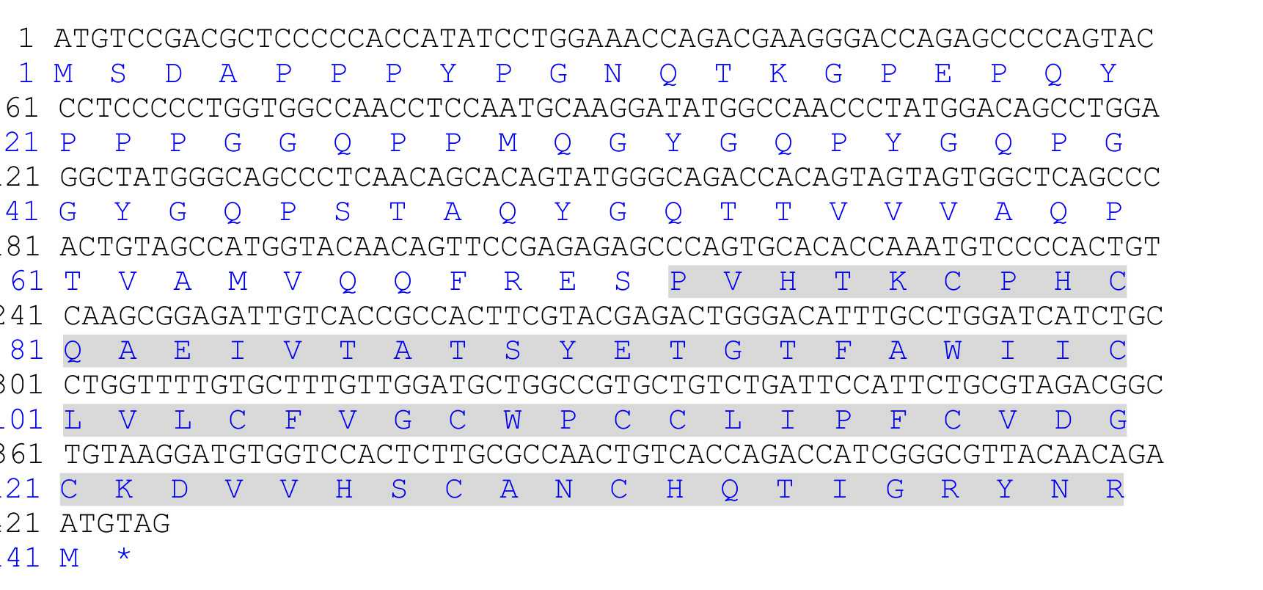
**

**Supplementary Figure 3.** The full-length cDNA and deduced amino acid sequence of P53 target protein1. The LITAF domains (amino acids 72-141) were shadowed.


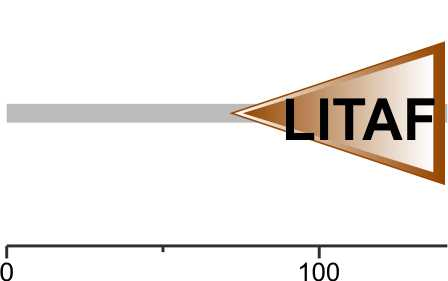


**Supplementary Figure 4.** Sequence analysis of P53 target protein1. The conserved domain of P53 target protein1 was analyzed by SMART. Schematic of the key functional domains of ChP53 target protein1 was shown above.

**
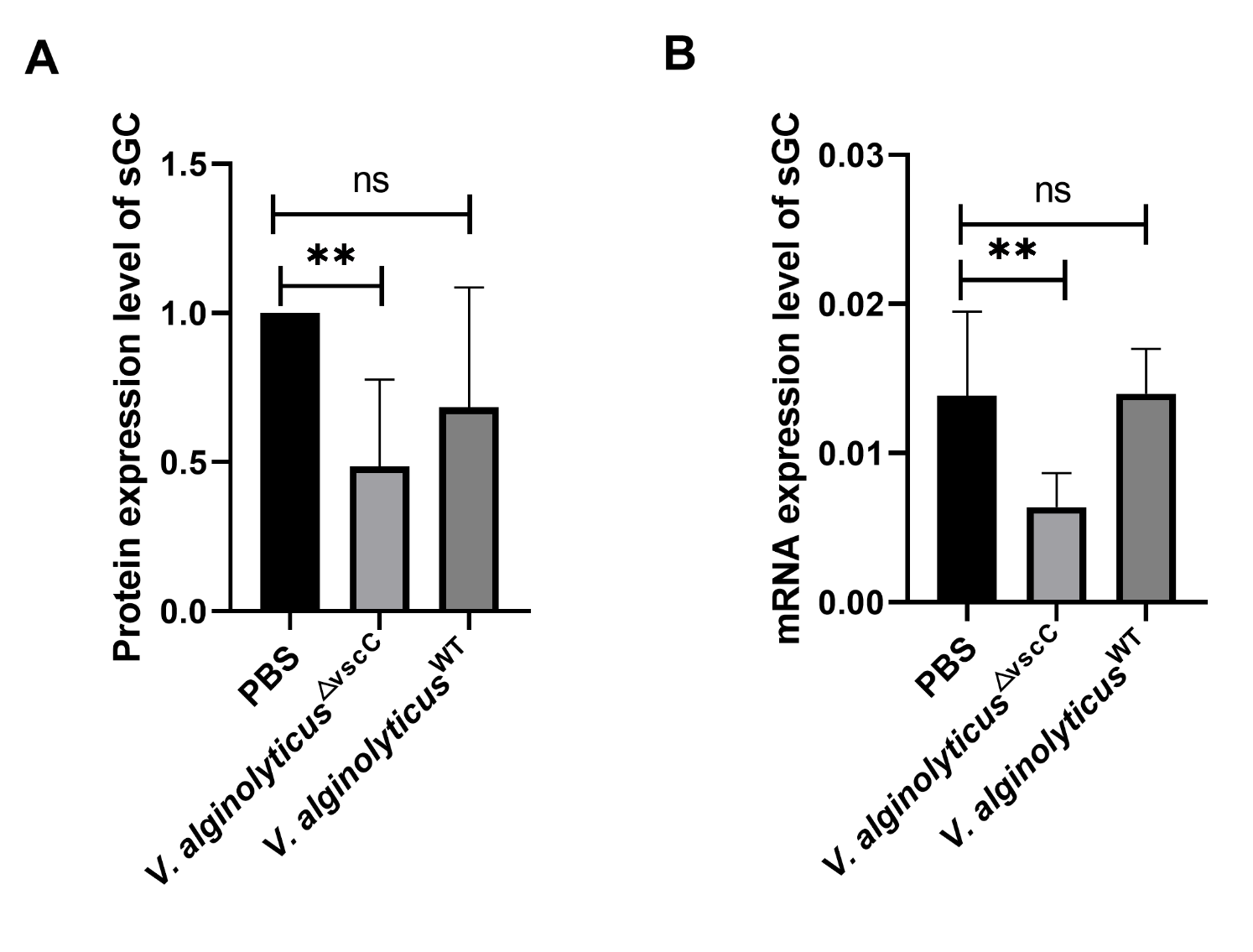
**

**Supplementary Figure 5. Relative expression level of *Ch*sGC protein. (A)** Relative expression of sGC at protein level. The expression level was set to 1 in the control group. Data were analyzed by paired t-test and presented as mean ± SEM (n = 3); **, p< 0.01. **(B)** Relative expression of sGC at mRNA level. The mRNA expression level of sGC was only significantly down-regulation post *V. alginolyticus*^△vscC^ strain infection, and no significant difference in *V. alginolyticus*^WT^ strain infection. mRNA levels were quantiﬁed by real-time PCR with GAPDH as a reference gene. Data were analyzed by unpaired t-test and presented as mean ± SEM (n = 3); **, p< 0.01.

**

**

**Supplementary Figure 6.** The standard curve of cGMP content. The standard curve of cGMP content was determined by 0 nM, 0.75 nM, 1.5 nM, 3 nM, 6 nM and 12 nM standard solution. Four-parameter Logistic curve fitting analysis was used for data statistical analysis.

**2. Supplementary Tables**

**Supplementary Table 1. Summary of protein identification results.** IBT quantitative protein analysis produced a total of 296,436 spectra, including 43,964 unique spectra. 16,510 peptides from 4,065 proteins were filtrated with FDR<=1% in total *C. hongkongensis* samples.

**Supplementary Table 2. Comparative analysis of identified proteins.** The identified proteins were annotated and classified into six groups according to up-regulated DEPs and down-regulated DEPs. A total of 107 proteins were differentially expressed following Vibrio injection, of which 68 were up-regulated and 39 down-regulated. Among these, three DEPs clusters were summerized, including common DEPs, *V. alginolyticus*^WT^-specific DEPs, and *V. alginolyticus*^△vscC^-specific DEPs.

**Supplementary Table 3. GO term enrichment.** GO term enrichment was performed to annotate and compare pathways induced in oysters injected with *V. alginolyticus*^WT^ and *V. alginolyticus*^△vscC^.

**Supplementary Table 4. Summary of Primers used in the study.**
